# Permeability of Magnetic Cores with Air Gaps

**DOI:** 10.3390/ma15031217

**Published:** 2022-02-06

**Authors:** Yuri N. Starodubtsev, Vladimir S. Tsepelev, Vladimir Ya. Belozerov, Viktor A. Zelenin

**Affiliations:** 1 Research Center for Physics of Metal Liquids, Ural Federal University, 620002 Yekaterinburg, Russia; iu.n.starodubtsev@urfu.ru; 2 Gammamet Research and Production Enterprise, 620131 Yekaterinburg, Russia; gammamet@gammamet.ru (V.Y.B.); zwa42523@mail.ru (V.A.Z.)

**Keywords:** permeability, FEMM simulation, nanocrystalline cut core, air gap, inductor

## Abstract

The influence of the geometric dimensions of the cut core and the number and size of air gaps on the effective permeability was investigated. Using dimensional analysis, an equation was obtained that relates the permeability of the cut core to the simplest dimensionless combination of the mean magnetic flux length *l*, single air gap length *l_g_*_1_, the cross-sectional area *S* of the core, and gap number *n_g_*. Permeability calculated from the geometric parameters of the cut core was compared with the effective permeability obtained using a two-dimensional FEMM simulation. Simulation has shown that the equation derived from dimensional analysis provides the best fit. The influence of each significant parameters *l*, *l_g_*_1_, *n_g_*, and *S* on cut core permeability is demonstrated. Experimental results have shown that an equation derived from dimensional analysis can be used to predict cut core permeability. In this case, one should take into account the residual air gap that forms after cutting the core. A method for assessing the residual air gap is proposed.

## 1. Introduction

Nanocrystalline cores are made from an amorphous metal tape with a thickness of about 20 μm. A tape with an amorphous structure is obtained by rapidly quenching a multicomponent melt on the surface of a rotating cooler drum [1]. To obtain a nanocrystalline structure, the tape-wound cores are annealed. Currently, a nanocrystalline soft magnetic material has been widely used that has a composition close to the classical Finemet Fe_73.5_Cu_1_Nb_3_Si_13.5_B_9_ [2]. In this alloy, Si and B contribute to the formation of an amorphous structure in rapidly quenched tape [3]. Copper provides fine copper-rich clusters, which create a chemical inhomogeneity of the amorphous matrix at the initial stage of annealing. These clusters are centers of nucleation of a large number of α-FeSi nanocrystals. Niobium inhibits the growth of the crystals to a higher temperature and provides a nanocrystalline structure with an average grain size of about 10 nm [4]. Instead of or together with Nb, other elements are used as inhibitors, for example, Mo, W, and V, which change the kinetics of heat release during crystallization [5] and contribute to obtaining the best magnetic properties during heat treatment of cores.

High permeability is associated with a weakening of the macroscopic magnetic anisotropy in the nanoscale structure of the material [6]. Iron-based nanocrystalline soft magnetic materials, in addition to high permeability, have high saturation magnetization. Nanocrystalline soft magnetic material has typical values of coercive force *H_c_* = 0.5 A·m^−1^, initial permeability μ*_i_* = 100,000, magnetic induction *B*_800_ = 1.3 T, and loss *P*_0.2/20,000_ = 0.4 W·kg^−1^. The combination of high permeability and saturation magnetization has provided widespread usage in power electronics [7,8] (pp. 277–345).

In a power electrical circuit, inductors are used to store and transmit electromagnetic energy. The magnetic energy of the core *W* (J) in an external magnetic field *H_e_* (A·m^−1^) is described as follows:

(1) $$W=\frac{BH_{e}}{2}V,$$

where *B* is the magnetic induction (T), and *V* is the core volume (m^3^). Magnetic induction and external magnetic field are related by the following relationship:

(2) $$B=\mu_{с}\mu_{0}H_{e},$$

where μ*_c_* is the core permeability, and μ_0_ is the magnetic constant (H·m^−1^). From (1) and (2), we obtain magnetic energy in the following form.

(3) $$W=\frac{B^{2}}{2\mu_{с}\mu_{0}}V.$$

Consequently, with a constant volume *V*, the magnetic energy stored in the inductor is greater in the core with high magnetic induction and low permeability. It is important to note that magnetic energy is higher in the core in which permeability is constant.

If an external magnetic field is created by winding with current *I* (A) and the number of turns *w*, then the external field *H_e_* can be found from Ampere’s circuital law:

(4) $$H_{e}l=Iw,$$

where *l* is the magnetic path length (m). Using Ampere’s law (4), we express magnetic energy in terms of electrical quantities:

(5) $$W=\frac{\mu_{с}\mu_{0}H_{e}^{2}}{2}V=\frac{\mu_{с}\mu_{0}Sw^{2}}{2l}I^{2}=\frac{LI^{2}}{2},$$

where *L* is the inductance (H):

(6) $$L=\frac{\mu_{с}\mu_{0}Sw^{2}}{l},$$

and the core volume is as follows:

(7) V=Sl,

where *S* is the cross-sectional area of a core (m^2^).

An inductor as an element of an electrical circuit is characterized by an inductance *L* and an electric current *I*. An inductor as a design element is characterized by the dimensions of the core *S* and *l*, the permeability of the core μ*_с_*, and the number of turns *w*. The design quantities are unknown, and they must be found in order to provide the specified values of *L* and *I*. From (2) and (4), we find the following.

(8) $$\frac{l}{\mu_{с}w}=\frac{\mu_{0}I}{B}.$$

After substituting (8) into (6), we obtain the following.

(9) $$Sw=\frac{LI}{B}.$$

Relations (8) and (9) represent a system of equations for determining the design parameters of the inductor. On the left side of the equations, there are unknown quantities, and on the right side, there are quantities that are determined by the design of the power circuit. Magnetic induction can be attributed to a limited specified value, since it is limited from the above by the saturation magnetization. Another limitation for *B* is the overheating temperature of the inductor, which is related core and winding losses. From (8) and (9), it follows that a decrease in magnetic induction results in an increase in the core’s size. It is impossible to unambiguously calculate the inductor; therefore, the successive approximation is used to optimize the inductor’s design.

An important advantage of nanocrystalline soft magnetic materials as an inductor core is low loss and high saturation magnetization. For the thermally limited design of the inductor, these advantages allow one to obtain a high working induction and a small core size.

Low permeability is obtained by creating air gaps in the core. In a closed core, the magnetic flux is concentrated in the magnetic material. Air gaps redistribute the magnetic flux so that part of it is scattered near the gap. Magnetic flux extends beyond the core cross-section, creating a fringing flux [9,10]. Effective permeability of the core with air gaps depends on geometric dimensions of the core (*S* and *l*), as well as on the single air gap length *l_g_*_1_ and the number of gaps *n_g_*. Since all these quantities strongly affect core losses [11,12,13], optimization of the number and size of air gaps in the inductor core is an important design problem.

## 2. Materials and Methods

Two-dimensional finite element modeling software FEMM 4.2 was used to simulate the effective permeability of annular core with air gaps [14]. A winding of 8 turns with a current of 3 A was uniformly applied to the annular core. The calculated effective permeability μ*_BH_* represented the ratio of the average normal magnetic induction *B_n_* in the magnetic cut core and in the same core filled with air. For the calculation, we used the magnetization curve of a composite core made of nanocrystalline soft magnetic alloy Fe_72.5_Cu_1_Nb_2_Mo_1.5_Si_14_B_9_ [8] (p. 265). In a composite core, all magnetic quantities are referred to the unit of the geometric cross-section of the core. The magnetic induction in a field of 800 A × m^−1^ is 0.83 T, the initial permeability is 40,000, and the maximum permeability is 380,000. For experimental studies, cut cores made of the same magnetic material were used. Experimental effective permeability μ*_L_* was calculated from the inductance of the winding uniformly applied to the core (see Formula (6)). Inductance was measured at 1 kHz using an E7-22 RLC meter (Chy Firemate Co., Ltd., Tainan City, Taiwan).

## 3. Theory

We will distinguish between core permeability μ*_c_* of the following:

(10) $$\mu_{с}=\frac{B}{\mu_{0}H_{e}},$$

and the permeability of the magnetic material μ.

(11) $$\mu =\frac{B}{\mu_{0}H_{i}}.$$

In Formulas (10) and (11), *H_e_* is the external magnetic field, and *H_i_* is the internal magnetic field. Thus, the quantity of μ determines the property of the magnetic material and the quantity of μ*_c_* determine the properties of the core, i.e., an object that has a well-defined geometry and is made of a magnetic material.

Internal and external fields are related by the following relation [15]:

(12) $$H_{i}=H_{e}-NM,$$

where *M* is magnetization (A·m^−1^), and *N* is the demagnetization factor. From (12), we find the following external field:

(13) $$H_{e}=H_{i}+N(\frac{B}{\mu_{0}}-H_{i}),$$

by considering the following:

(14) $$B=\mu_{0}(M+H_{i}),$$

and substitute (13) in (10).

(15) $$\mu_{с}=\frac{B}{\mu_{0}H_{i}+N(B-\mu_{0}H_{i})}=\frac{\mu }{1+N(\mu -1)}.$$

If material permeability μ is much greater than 1, then (15) can be reduced to the following form.

(16) $$\frac{1}{\mu_{с}}=\frac{1}{\mu }+N.$$

It follows from (16) that, in the limit μ → ∞, core permeability is inversely proportional to the demagnetization factor *N*, i.e., μ*_c_* does not depend on the magnetic material.

Let us calculate the demagnetization factor *N* of an annular core with a magnetization *M*, which has *n_g_* gaps of length *l_g_*_1_ each. Let us assume that the magnetic flux in the magnetic material and in the air gaps is uniform, and there is no electric current. If we choose a circle of length *l* as contour, then, taking into account the magnetic field in the magnetic material −*NM* and in air gap *M*, from (4), we obtain the following.

(17) $$-NM(l-n_{g}l_{g1})+Mn_{g}l_{g1}=0.$$

From (17), we find the demagnetization factor of a magnetic contour of length *l* with cuts:

(18) $$N=\frac{l_{g}}{l-l^{g}},$$

where *l_g_* = *n_g_l_g_*_1_ represent the total air gap. Since Formula (18) was obtained for a contour, it is valid for a thin annulus in which the cross section is *S* → 0, with uniform magnetization inside the ferromagnet (the density of volume magnetic charges is zero) and the absence of magnetic scattering near the air gap, that is, for a small air gap when *l_g_* << *l*.

After substituting (18) into (15), we obtain the following.

(19) $$\mu_{c}=\frac{\mu }{1+\left(\mu -1\right)\frac{l_{g}}{l-l_{g}}}.$$

**Figure 1:**
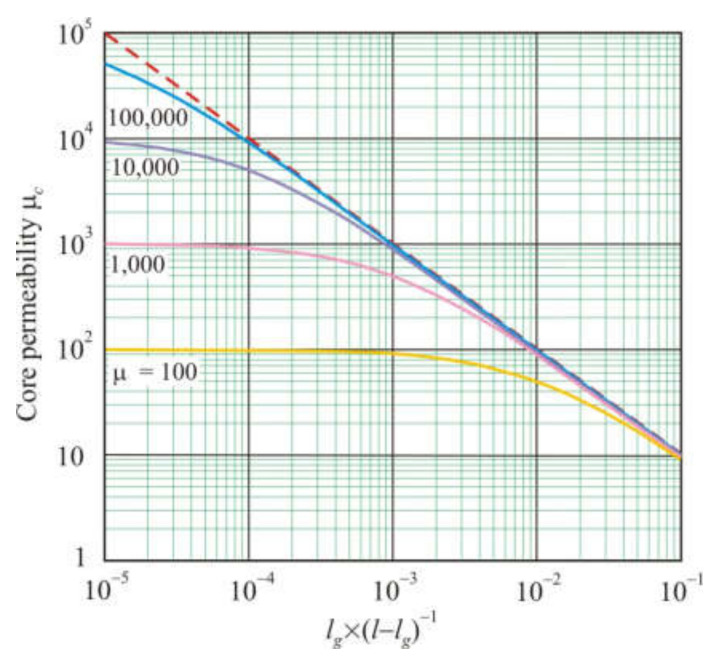
Dependences of the annular core permeability μ*_с_* on the *l_g_* × (*l* − *l_g_*)^−1^ ratio for magnetic material with different permeability μ. The dashed line corresponds to equation μ*_с_*= *l_g_* × (*l* − *l_g_*)^−1^.

Figure 1 shows the dependences of the core permeability μ*_c_* on the ratio of the air gap length *l_g_* to the length of the magnetic part of the core (*l* − *l_g_*), calculated by formula (19). The dashed straight line in Figure 1 corresponds to the following equation.

(20) $$\mu_{c}=\frac{l-l_{g}}{l_{g}}.$$

Starting from a certain value *l_g_* × (*l* − *l_g_*)^−1^, core permeability depends only on geometric parameters. For *l_g_* << *l*, instead of (20), one can use the following equation.

(21) $$\mu_{c}=\frac{l}{l_{g}}.$$

In order to determine the relationship between the permeability and the geometric parameters of the cut core, we will use dimensional analysis. Core permeability μ*_c_* is a dimensionless quantity that decreases with an increase in the number of gaps, *n_g_*, the length of single gap, *l_g_*_1_, the cross-sectional area of the core *S*, and also with a decrease in the mean magnetic path length, *l*. This makes it possible to relate core permeability μ*_c_* with the simplest dimensionless combination of these significant geometric quantities in the following form.

(22) $$\mu_{с}=\frac{l}{n_{g}l_{g1}}+\frac{l}{n_{g}\sqrt{S}}.$$

Equation (22) can also be written in the following form:

(23) $$\mu_{с}=\frac{l}{l_{g}}\left(1+\frac{l_{g}}{n_{g}\sqrt{S}}\right)$$

for independent variables *l*, *l_g_*, *n_g_*, and *S*, where *l_g_* = *l_g_*_1_*n_g_* is the total air gap, and also in the following form:

(24) $$\mu_{с}=\frac{l}{l_{g}}\left(1+\frac{l_{g1}}{\sqrt{S}}\right)$$

for independent variables *l*, *l_g_*, *l_g_*_1_, and *S*.

To determine the permeability of a cut core, designers usually use the following relationship [9,10]:

(25) $$\mu_{c}=F\frac{l}{l_{g}},$$

where *F* is the fringing flux factor:

(26) $$F=1+\frac{l_{g}}{n_{g}\sqrt{S}}\ln\left(\frac{l_{w}}{l_{g}}\right),$$

and *l_w_* is the length of the winding applied to the core. If the winding is applied uniformly along the entire length of the core, then *l_w_* = *l*. Equation (25) was first obtained by Partridge [16].

Next, we will evaluate the applicability of Equations (21), (22), and (25) for calculating the permeability of cut cores.

## 4. Simulation

**Figure 2:**
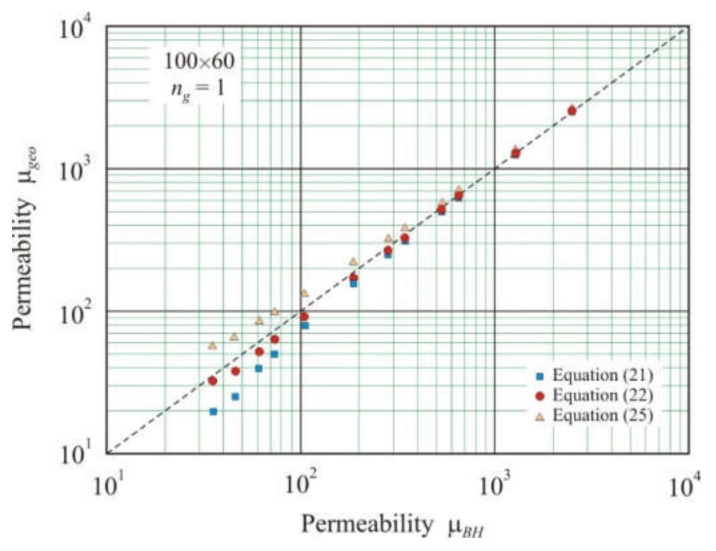
Relationship between geometric permeability μ*_geo_* calculated by Equations (21), (22), and (25) and effective permeability μ*_BH_* obtained using FEMM simulation in soft magnetic annuluses 100 × 60 mm with one air gap of length *l_g_*_1_ from 1 × 10^−4^ to 1.28 × 10^−2^ m. The dashed line corresponds to equation μ*_geo_*= μ*_BH_*.

Figure 2 shows the relationship of the permeability μ*_geo_* calculated from Equations (21), (22), and (25), which includes only the geometric parameters of the core, with effective permeability μ*_BH_* obtained using two-dimensional FEMM simulations. Annular cores 100 × 60 mm in size had one air gap with a length of 1 × 10^−4^ to 1.28 × 10^−2^ m. The mean magnetic path length was taken as follows:

(27) $$l=\frac{\pi \left(D_{o}+D_{i}\right)}{2},$$

where *D_e_* is the outer diameter, and *D_i_* is the inner diameter of core. The effective magnetic path length *l_ef_* is determined by the following formula.

(28) $$l_{ef}=\frac{\pi \left(D_{o}-D_{i}\right)}{\ln\frac{D_{o}}{D_{i}}}.$$

For a 100 × 60 core, Formulas (27) and (28) provide magnetic path lengths that differ by only 2%.

As a cross-section area of the core for two-dimensional modeling, we used the following.

(29) $$\sqrt{S}=\frac{D_{o}-D_{i}}{2}.$$

For a planar core, *l_g_*_1_ × *S*^−0.5^ in Equation (24) represents the ratio of the length of a single air gap to its width.

From Figure 2, it follows that, at a small gap (high permeability), the geometric permeability μ*_geo_* is close for all three Equations (21), (22), and (25), and these values are in good agreement with effective permeability μ*_BH_*. As the gap increases, the discrepancy between the different equations increases. Partridge Equation (25) overestimates permeability, and simple ratio (21) underestimates. Equation (22) obtained from dimensional analysis has the smallest deviation from the straight line μ*_geo_* = μ*_BH_*. This linear relationship on the logarithmic scale has a highly adjusted coefficient of determination *R*^2^*_adj_* = 0.9995. The deviation from the straight line μ*_geo_* = μ*_BH_* characterizes the exponent *s* = 1.04 in the power function.

(30) $$\mu_{geo}\propto \mu_{BH}^{s}.$$

**Figure 3:**
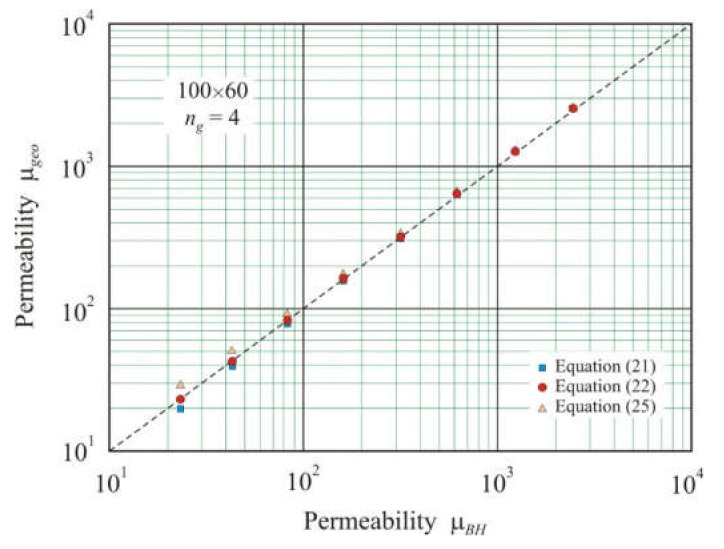
Relationship between geometric permeability μ*_geo_* calculated by Equations (21), (22), and (25) and effective permeability μ*_BH_* obtained using FEMM simulation in soft magnetic annuluses 100 × 60 mm with four air gaps and total length *l_g_* from 1 × 10^−4^ to 1.28 × 10^−2^ m. The dashed line corresponds to equation μ*_geo_*= μ*_BH_*.

As the number of gaps increases, the convergence of μ*_geo_* and μ*_BH_* improves. Figure 3 shows the relationship between the geometric permeability μ*_geo_*, calculated from Equations (21), (22), and (25), and the effective permeability μ*_BH_* for annular cores of 100 × 60 mm with four air gaps. The best convergence is shown by Equation (22) with the coefficient of determination *R*^2^*_adj_* = 1 and exponent *s* = 1.01.

**Figure 4:**
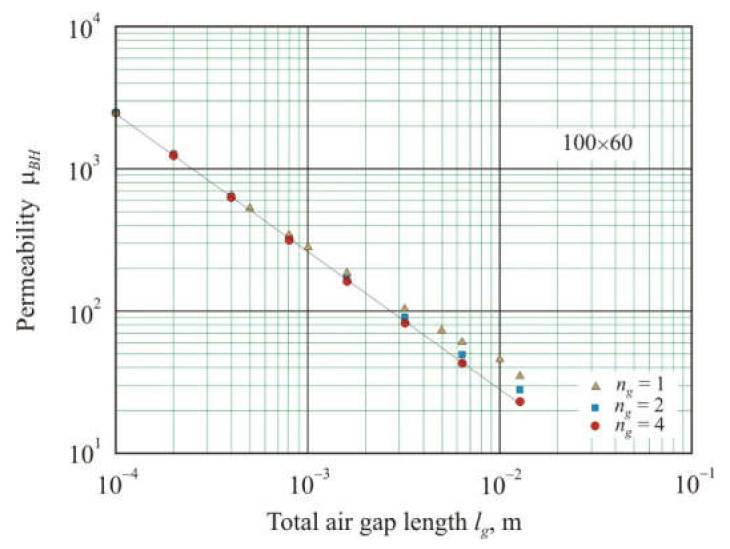
Relationship of effective permeability μ*_BH_* with the total length of air gaps *l_g_* in soft magnetic annuluses of 100 × 60 mm with the number of air gaps *n_g_* = 1, 2, and 4.

If we take cores with the same *l*, *S,* and *n_g_*, then, in accordance with (22), core permeability should be reciprocal relative to total air gap *l_g_*^−1^. Figure 4 shows the dependence of the effective permeability μ*_BH_* on *l_g_* for 100 × 60 cores with *n_g_* = 1, 2, and 4. It can be seen that for cores with four gaps, the dependence on the logarithmic scale is linear with the coefficient of determination *R*^2^*_adj_* = 0.9997 and the exponent *s* = −0.97 for power function (30). With a decrease in the number of gaps, nonlinearity increases, and it is particularly significant for large *l_g_*. Nonlinearity is affected by the second term of the sum in Equation (22), which increases with a decreasing number of gaps.

**Figure 5:**
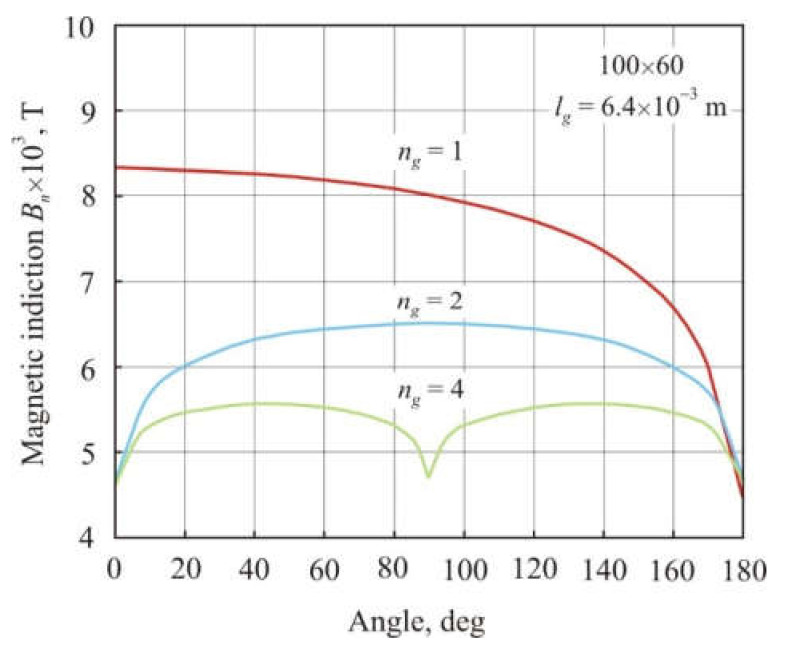
Distribution of normal magnetic induction *B_n_* averaged over the cross-section of an annular core 100 × 60 mm depending on the angular coordinate. The cores had the same total air gap length *l_g_* = 6.4 × 10^–3^ with the number of gaps *n_g_* = 1, 2, and 4.

Figure 5 shows the distribution of the normal magnetic induction *B_n_* averaged over the cross-section of a 100 × 60 annular core depending on the angular coordinate. Cores with *n_g_* = 1, 2 and 4 air gaps had the same total air gap length *l_g_* = 6.4 × 10^−3^ m. The smallest magnetic flux density in Figure 5 corresponds to the middle of the air gap. It can be seen that with an increase in the number of gaps, magnetic flux becomes more uniform along the length of the core, and at the same time, average magnetic induction and, consequently, permeability decrease.

**Figure 6:**
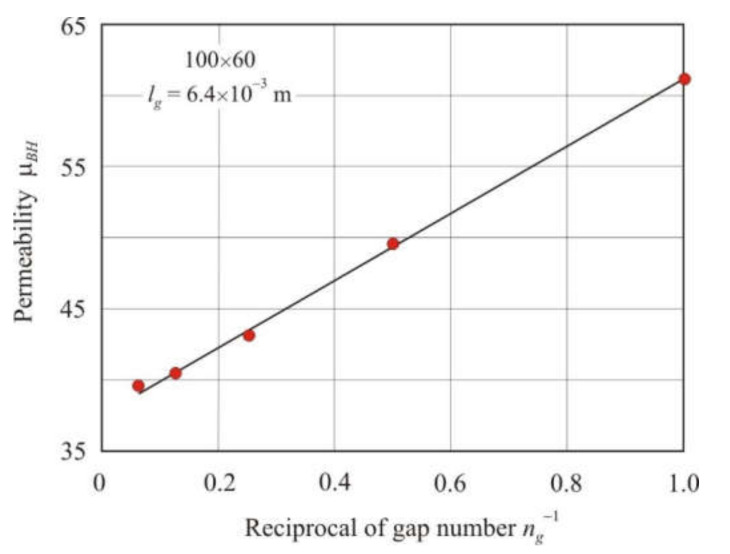
Dependence of the effective permeability μ*_BH_* on the reciprocal of air gap number *n_g_*^−1^ in soft magnetic annuluses 100 × 60 mm with the total air gap length *l_g_* = 6.4 × 10^−3^ m.

The decrease in permeability with an increase in the number of gaps with constant *l*, *S,* and *l_g_* follows from Equation (22). Figure 6 shows the dependence of the effective permeability μ*_BH_* on the reciprocal of air gap number *n_g_*^−1^ in annular cores of 100 × 60 mm with a total air gap length *l_g_* = 6.4 × 10^−3^ m. The number of gaps in the cores *n_g_* = 1, 2, 4, 8, and 16. The dependence of μ*_BH_* on *n_g_*^−1^ is linear with the coefficient of determination *R*^2^*_adj_* = 0.998.

**Figure 7:**
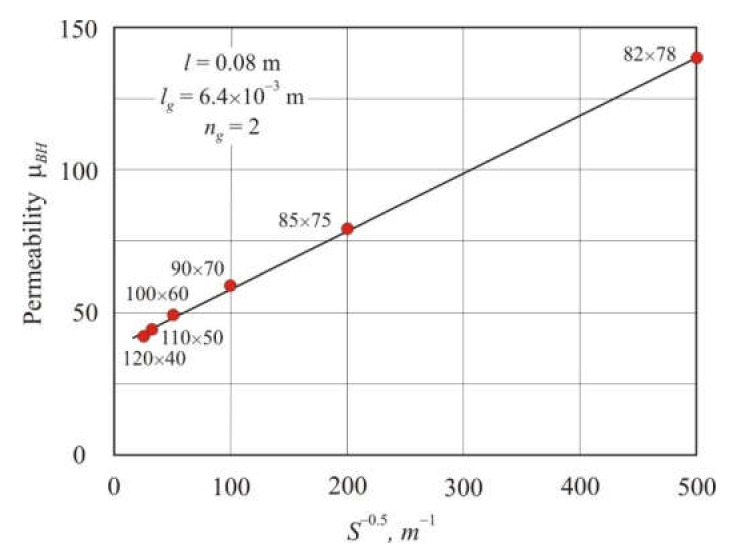
Dependence of the effective permeability μ*_BH_* on *S*^−0.5^ in soft magnetic annuluses with two air gaps, the mean magnetic flux length *l* = 0.08 m and the total air gap length *l_g_* = 6.4 × 10^−3^ m. Core dimensions are shown near points.

From Equation (22), it follows that with constant *l*, *l_g_*, and *n_g_*, core permeability is linearly related to *S*^−0.5^, where *S* is the cross-section area of the core. Figure 7 shows the dependence of the effective permeability μ*_BH_* on *S*^−0.5^ in annular cores with two air gaps, the mean magnetic path length *l* = 0.08 m, and the total air gap length *l_g_* = 6.4 × 10^−3^ m. The dependence of μ*_BH_* on *S*^−0.5^ is linear with the coefficient of determination being *R*^2^*_adj_* = 0.998.

**Figure 8:**
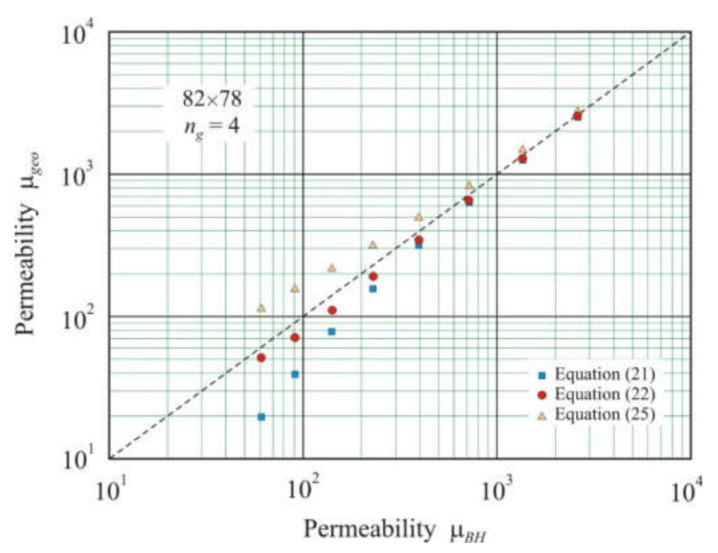
Relationship between geometric permeability μ*_geo_* calculated by Equations (21), (22), and (25) and effective permeability μ*_BH_* obtained using FEMM simulation in soft magnetic annuluses 82 × 78 mm with four air gaps and total length *l_g_* from 1 × 10^−4^ to 1.28 × 10^−2^ m. The dashed line corresponds to equation μ*_geo_*= μ*_BH_*.

Comparing cores with different cross-section areas allows us to return again to Equations (21), (22), and (25). Figure 8 shows the relationship between geometric permeability μ*_geo_*, calculated from Equations (21), (22), and (25), and effective permeability μ*_BH_* for 82 × 78 mm annular cores with four air gaps and the total air gap length from 1 × 10^−4^ to 1.28 × 10^−2^ m. Comparison of cores 100 × 60 mm (Figure 3) and 82 × 78 mm (Figure 8), which have the same *l*, *l_g_*, and the number of gaps *n_g_* = 4 but different cross-section areas shows that in a core with a small cross-section, the convergence of geometric permeability μ*_geo_* with effective permeability μ*_BH_* is noticeably worse. Despite this, Equation (22) again provides the best results with the coefficient of determination *R*^2^*_adj_* = 0.998 and exponent *s* = 1.02 for the power function.

**Figure 9:**
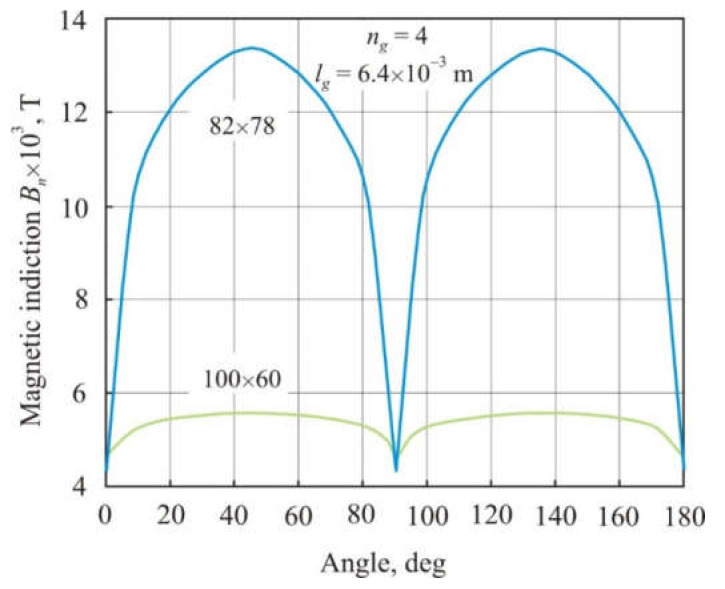
Distribution of normal magnetic induction *B_n_* averaged over the cross-section of annular cores 100 × 60 mm and 82 × 78 mm depending on the angular coordinate. The cores have the same mean magnetic path length *l* = 0.08 m, the total air gap length *l_g_* = 6.4 × 10^−3^ m, and the number of gaps *n_g_* = 4.

Figure 9 shows the distribution of normal magnetic induction *B_n_* averaged over the cross-section of annular cores 100 × 60 mm and 82 × 78 mm depending on the angular coordinate. The cores were in an external magnetic field of 97 A × m^−1^. They have the same *l* = 0.08 m, *l_g_* = 6.4 × 10^−3^ m, and the number of gaps *n_g_* = 4 but differ in the ratio *l* × *S*^−0.5^. Four air gaps divide the core into four magnetic pieces. The *l* × *S*^−0.5^ ratio in the 82 × 78 mm core is 10-times greater than in the 100 × 60 mm core. It follows from Figure 8 that the magnetic induction in the 82 × 78 mm core is much higher and is more nonuniformly distributed along the length of the core. This is consistent with the results of calculating the demagnetizing factor *N* for rectangular prisms [17], which decreases with an increase in ratio *l* × *S*^−0.5^. A decrease in *N* promotes the convergence of the internal and external magnetic fields and an increase in magnetic induction and core permeability (see Equation (16)).

## 5. Experiment

After cutting the nanocrystalline core in half and then compressing the two halves tightly, core permeability decreases. This indicates that a residual air gap has formed in the core. The length of the residual air gap *l_o_* can be found by taking into account that after tight compression, condition *l_o_* << *l* is satisfied. In this case, from (21), we have the following:

(31) $$l_{o}=\frac{l}{\mu_{L}},$$

where μ*_L_* is the measured permeability of the core after cutting and tight compression. For a more accurate determination of the residual air gap length, Equation (23) can be used, from which the following is obtained.

(32) $$l_{o}=\frac{l}{\mu_{L}-\frac{l}{n_{g}\sqrt{S}}}.$$

For most of the cores in this study, Equations (31) and (32) produce similar results.

**Figure 10:**
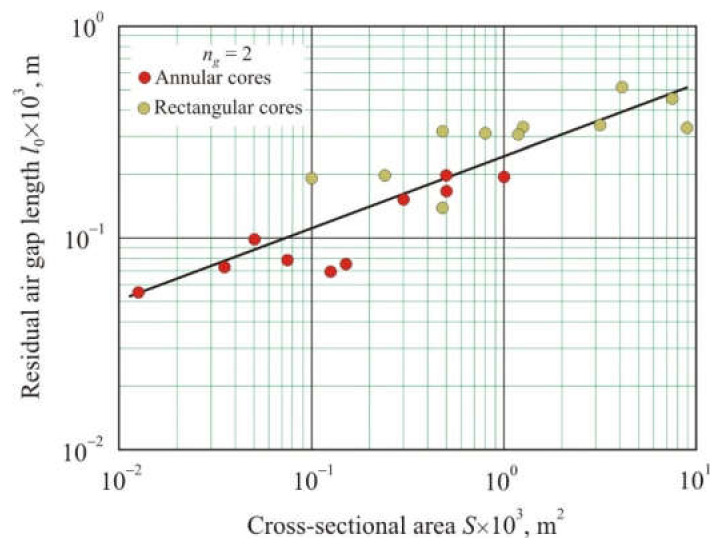
Dependence of the residual air gap length *l_o_* on the cross-sectional area *S* after cutting the nanocrystalline core in half and then tightly compressing the two halves.

Figure 10 shows the dependence of the residual air gap length *l_o_* on cross-sectional area *S* after cutting various nanocrystalline cores in half and then tightly compressing the two halves. The residual air gap length was calculated from Equation (32). It follows from Figure 10 that with an increase in the cross-section area, the residual air gap length grows. The linear relationship between *l_o_* and *S* on a logarithmic scale has a coefficient of determination *R*^2^*_adj_* = 0.78 and can be represented as a power function:

(33) $$l_{o1}=0.00125\times S^{0.34},$$

where *l_o_*_1_ is the single residual air gap length.

**Figure 11:**
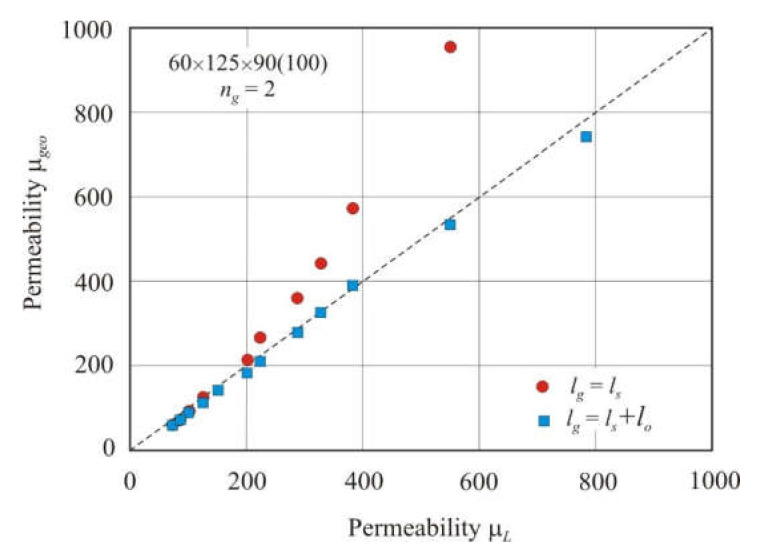
Relationship of geometric permeability μ*_geo_* and measured permeability μ*_L_* in a 60 × 125 × 90 (100) rectangular core with two air gaps. The total air gap length *l_g_* was taken equal to *l_s_* or (*l_s_* + *l_o_*), where *l_s_* is the spacer thickness, and *l_o_* is the residual air gap length. The dashed line corresponds to equation μ*_geo_*= μ*_L_*.

**Figure 12:**
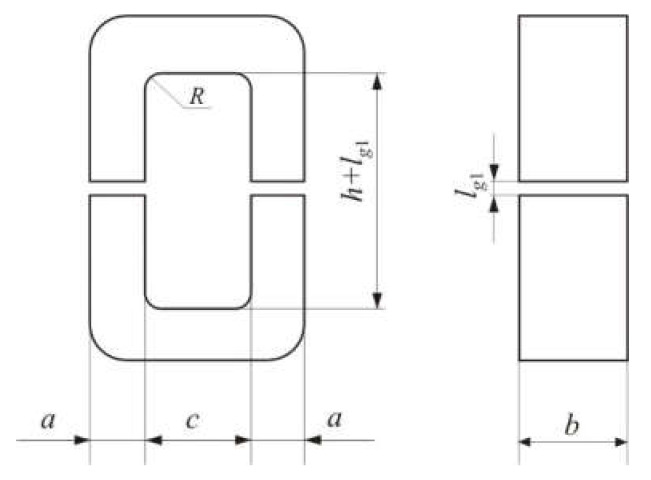
Dimensions of a rectangular core with a general dimensional formula *a* × *b* × *h* (*c*).

Figure 11 shows the effect of the residual air gap on the permeability of the cut core. The 60 × 125 × 90 (100) rectangular core had two air gaps. Figure 12 shows the dimensions of a rectangular core with a general dimensional formula *a* × *b* × *h* (*c*). Spacers were inserted into the air gaps. The spacer thickness *l_s_* was varied from 0.3 to 9.8 mm. The geometric permeability of the core μ*_geo_* was calculated using Equation (23). The total air gap length *l_g_* was taken equal to *l_s_* or (*l_s_* + *l_o_*). The total residual air gap *l_o_* = 0.454 mm was calculated using Equation (32). Geometric permeability μ*_geo_* was compared with measured permeability μ*_L_*. Figure 11 shows that the permeability prediction accuracy of the cut core is significantly improved when the residual air gap is taken into account. If the fraction of the residual air gap is small, then the calculated and measured permeabilities are in good agreement.

**Figure 13:**
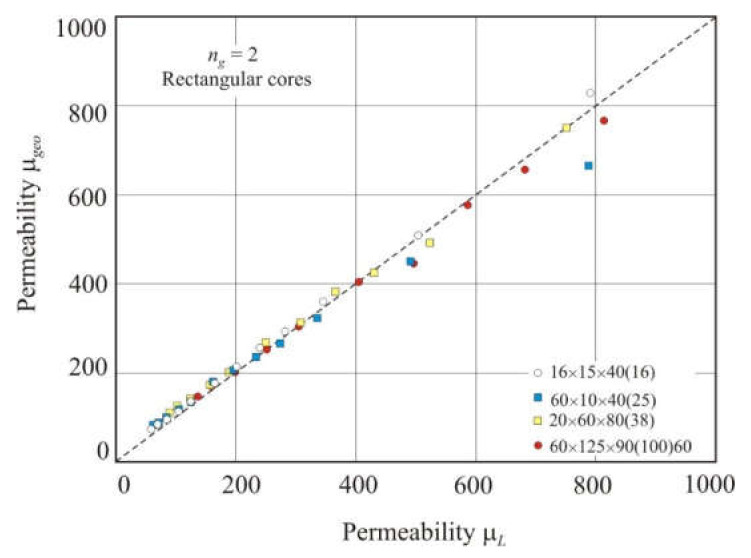
Relationship between geometric permeability μ*_geo_* and measured permeability μ*_L_* in nanocrystalline rectangular cores with two air gaps. The total air gap length *l_g_* was taken equal to (*l_s_* + *l_o_*), where *l_s_* is the spacer thickness, and *l_o_* is the residual air gap length. The dashed line corresponds to equation μ*_geo_*= μ*_L_*.

Figure 13 shows the relationship of geometric permeability μ*_geo_* with the measured permeability μ*_L_* in different nanocrystalline rectangular cores with two gaps. Permeability μ*_geo_* was calculated using Equation (23). Spacers were inserted into the gaps, and the total air gap length *l_g_* was taken equal to (*l_s_* + *l_o_*). The linear relationship between the calculated and measured permeabilities has a high coefficient of determination *R*^2^*_adj_* = 0.993 and an exponent *s* = 0.96 in the power function.

**Figure 14:**
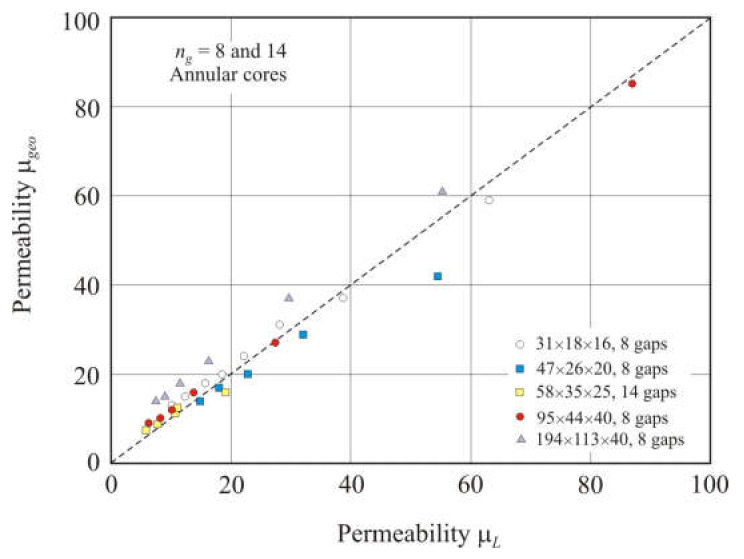
Relationship between geometric permeability μ*_geo_* and measured permeability μ*_L_* in nanocrystalline annular cores with 8 or 14 air gaps. The total air gap length *l_g_* was taken equal to (*l_s_* + *l_o_*), where *l_s_* is the spacer thickness, and *l_o_* is the residual air gap length. The dashed line corresponds to equation μ*_geo_*= μ*_L_*.

Figure 14 shows the relationship of the geometric permeability μ*_geo_* with the measured permeability μ*_L_* in different nanocrystalline annular cores with 8 or 14 air gaps. Spacers were inserted into the gaps, and the air gap was equal to the sum (*l_s_* + *l_o_*). The linear relationship between the calculated and measured permeabilities was worse than in the cores with two gaps. The coefficient of determination *R*^2^*_adj_* of this linear dependence is 0.96, and the exponent is *s* = 0.90 in the power function.

Experimental results demonstrate that Equation (22) can be used to accurately predict the permeability of a cut core. In practice, an account should be taken of the residual air gap that forms after the core is cut. A method for estimating the residual air gap length is proposed. The method takes into account that the scattering of the magnetic flux will be minimal when individual magnetic pieces of the cut core are tightly compressed.

## 6. Conclusions

Cut core permeability is a dimensionless quantity, which decreases with an increase in the number of air gaps *n_g_*, single gap length *l_g_*_1_, cross-sectional area *S* of the core, and also with a decrease in the mean magnetic flux length *l*. Using dimensional analysis, an equation was obtained that relates cut core permeability to the simplest dimensionless combination of these significant geometric quantities. The equation has a simpler form in comparison with the traditional Partridge equation.

Permeability μ*_geo_*, calculated from the geometric parameters of the cut core, was compared with the effective permeability μ*_BH_* obtained using a two-dimensional FEMM simulation. Simulation has shown that the equation derived from dimensional analysis provides the best fit for μ*_geo_* and μ*_BH_*. As the gap number increases, the convergence of these permeabilities improves. The influence of each of the significant parameters *l*, *l_g_*_1_, *n_g_*, and *S* on the cut core permeability is demonstrated. It is shown that significant geometric parameters affect permeability according to an equation derived from dimensional analysis.

Experimental results have shown that an equation relating permeability to geometric parameters can be used to predict the cut core permeability. In practice, account should be taken on the residual air gap that forms after the core is cut. A method for estimating the residual air gap length is proposed. The method takes into account that the scattering of the magnetic flux will be minimal when individual magnetic pieces of the cut core are tightly compressed.

## Data Availability

The data presented in this article are available upon request from the corresponding author.
